# Book Review: “Ultrasound for the Generalist: A Guide to Point-of-Care Imaging”

**DOI:** 10.24908/pocus.v7i2.15765

**Published:** 2022-11-21

**Authors:** Daniel Restrepo

**Affiliations:** 1 Department of Medicine, Massachusetts General Hospital, Harvard Medical School Boston, MA USA

**Keywords:** POCUS, Education, General Medicine

**Ultrasound for the Generalist: A Guide to Point-of-Care Imaging**

Edited by Sarb Clare and Chris Duncan

Cambridge University Press: Cambridge, UK 2022

ISBN: 978-1-108-79707-8

256 Pages

$50.78

Drs. Clare and Duncan, as well as the contributing authors for the chapters have compiled a comprehensive and complete work that champions the practice of point-of-care ultrasound (POCUS) by generalists, denoting its myriad utilities and diagnostic power. While a sizeable portion of the book is dedicated to the more traditional applications for generalists such as thoracic, abdominal, cardiac, and vascular ultrasound, the work spans far beyond this with robust chapters on musculoskeletal, pelvic, and obstetric applications. Furthermore, they go on to denote the utility of POCUS in arenas outside of the brick-and-mortar acute care hospital with chapters dedicated to ultrasound within rural medicine, hospital at home and palliative medicine. 

The book begins with a thorough and complete compendium of all the physics, machine basics and imaging modalities needed to begin learning POCUS. As a frequent teacher of ultrasound, I found their basics chapter to have some of the most accessible explanations for inherently complicated topics that I have ever encountered. Complex physics and artifacts were well-deconstructed with explanations to artifacts that I had previously struggled to explain to my own learners. After this, they demonstrate a consistent pattern of beginning with first principles accessible to beginners and building from there within each chapter. This is most apparent in their chapter on cardiac ultrasound wherein they conclude with advanced views and measurements that are instructive to even the most advanced ultrasound practitioners. The book is accompanied by an online companion with learning videos to complement the content featured in the text, however despite showing stills in the print, the annotation and quality of the images makes it so learning can occur despite presenting an inherently dynamic concept in static form. 

Throughout the work, the authors strive to integrate the practice of POCUS with the clinical reasoning and the traditional physical exam. Indeed, their syndrome-based approach to a variety of clinical scenarios strongly underscores the utility of ultrasound: a means to augment our diagnostic prowess at the bedside by complementing the history and traditional physical examination without sacrificing time. In many of the chapters, content is incredibly well-organized into charts or figures that allow for cognitive chunking such as cardiac vs. non-cardiac causes of B-lines in lung ultrasound or accompanying images of consolidation with the patient’s attendant radiography for better spatial understanding. 

In future editions, the book would benefit from learning objectives in each chapter as well as intentional divisions of the material into introductory and advanced sections as certain readers may be intimidated by more difficult topics such as Doppler imaging. Additionally, several strong claims are made throughout and while some of these may be grounded in evidence, without references the reader may be tempted to assume they simply represent opinion. Though I share in their excitement for the generalist to use the entire extent of their skills to diagnose their patient, at times the zeal espoused by the authors can overshadow the importance of knowing when comprehensive imaging is needed. 

Overall, this represents a valuable work in illustrating the role of the generalist as a bedside diagnostician and how they can augment their ability to make diagnoses in real-time. This book serves as a useful reference for general medicine practitioners ranging from those who are newly acquiring the skill to seasoned point-of-care ultrasound teachers. 

## Disclosures

None.

